# Estrogen attenuates the spondyloarthritis manifestations of the SKG arthritis model

**DOI:** 10.1186/s13075-017-1407-9

**Published:** 2017-09-07

**Authors:** Hyemin Jeong, Eun-Kyung Bae, Hunnyun Kim, Yeong Hee Eun, In Young Kim, Hyungjin Kim, Jaejoon Lee, Chan Hong Jeon, Eun-Mi Koh, Hoon-Suk Cha

**Affiliations:** 1 Division of Rheumatology, Department of Internal Medicine, Soonchunhyang University Hospital, Bucheon, South Korea; 2 Samsung Biomedical Research Institute, Seoul, South Korea; 3 Division of Rheumatology, Department of Internal Medicine, Samsung Medical Center, Sungkyunkwan University School of Medicine, 81 Irwon-Ro, Gangnam-gu, Seoul, 06351 South Korea

**Keywords:** Estrogen, Spondyloarthritis, Mice

## Abstract

**Background:**

Ankylosing spondylitis (AS) is a male-predominant disease, and radiographic evidence of damage is also more severe in males. Estrogen modulates immune-related processes such as T cell differentiation and cytokine production. This study aimed to evaluate the effect of estrogen on the disease activity of spondyloarthritis (SpA).

**Methods:**

The effects of estrogen on the development of arthritis were evaluated by performing ovariectomy and 17β-estradiol (E2) pellet implantation in zymosan-treated SKG mice. Clinical arthritis scores were measured, and ^18^F-fluorodeoxyglucose (^18^F-FDG) small-animal positron emission tomography/computed tomography performed to quantify joint inflammation. The expression of inflammatory cytokines in joint tissue was measured.

**Results:**

E2-treated mice showed remarkable suppression of arthritis clinically and little infiltration of inflammatory cells in the Achilles tendon and intervertebral disc. ^18^F-FDG uptake was significantly lower in E2-treated mice than in sham-operated (sham) and ovariectomized mice. Expression of TNF, interferon-γ, and IL-17A was significantly reduced in E2-treated mice, whereas expression of sclerostin and Dickkopf-1 was increased in E2-treated mice compared with sham and ovariectomized mice.

**Conclusions:**

Estrogen suppressed arthritis development in SKG mice, a model of SpA. Results of this study suggest that estrogen has an anti-inflammatory effect on the spondyloarthritis manifestations of the SKG arthritis model.

## Background

Ankylosing spondylitis (AS) is a chronic inflammatory disease that primarily affects the sacroiliac joints and spine. Syndesmophytes and ankylosis occur as a consequence of longstanding inflammation, resulting in loss of function and disability. AS is a male-predominant disease and men with this disease also tend to have radiographic evidence of more severe damage [1]. Diagnosing this disease at an early stage is challenging; the classification criteria for axial spondyloarthritis (SpA) were developed by the Assessment of SpondyloArthritis international Society (ASAS) [2] and the ASAS criteria therefore cover both patients with or without established radiographic changes in the sacroiliac joint. The clinical presentation, clinical disease activity, and treatment responses of both groups are similar [3, 4]. However, patients with non-radiographic axial spondyloarthritis (nr-axSpA) have lower C-reactive protein (CRP) [5, 6] and less active inflammation on magnetic resonance imaging (MRI) than patients with AS [6]. Interestingly, the male-to-female ratio of nr-axSpA has been reported to be approximately 1:1, whereas it is 2:1 in patients with AS [5–7]. The proportion of female patients was significantly lower among the group of patients who progressed from nr-axSpA to AS [8]. The female predominance of patients with nr-axSpA can be understood in the same context, as male patients with AS have more severe radiographic damage than female patients with AS [1, 9]. The underlying pathogenesis of the differences between men and women with AS is still unknown.

Jimenez-Balderas et al. reported that 17β-estradiol (E2) levels are lower in patients with active AS than in those with inactive AS. Peripheral arthritis was shown to subside and the clinical activity of AS was reduced after oral estrogen therapy [10]. Estrogen modulates immune-related processes, such as T cell differentiation and cytokine production [11]. Estrogen is involved in the immune response, characterized by a bias toward type 2 cytokine profiles [12]. Animal model studies have suggested that estrogen can inhibit the differentiation of T helper (Th)17 cells from naïve T cells [13, 14].

Wnt proteins play an important role in the anabolic pattern of joint remodeling in AS. Dickkopf-related protein 1 (Dkk1) is an inhibitor of the Wnt pathway and acts as a master regulator of joint remodeling [15]. Increased Dkk1 is related to bone resorption, whereas decreased Dkk1 is linked to new bone formation [16]. To the best of our knowledge, there are no data about the relationship between estrogen and Wnt inhibitors in patients with AS.

Sakaguchi et al. characterized the SKG mouse strain, which develops spontaneous autoimmune inflammatory arthritis after systemic ß-glucan exposure [17]. Although the SKG mouse model was first used as a rheumatoid arthritis (RA) model, Ruutu et al. reported that SKG mice have clinical characteristics of SpA, including spondylitis, enthesitis, and bowel inflammation [18]. The role of estrogen in the pathogenesis and disease activity of SpA has not been determined. We hypothesized that estrogen has an anti-inflammatory effect on the disease activity of SpA. Therefore, the aim of this study was to investigate the role of estrogen in the disease activity of SpA using the SKG mouse model. We evaluated the effect of estrogen on the development and progression of SpA and investigated the relationship between estrogen and inflammatory cytokines.

## Methods

### Experimental animals

SKG mice purchased from CLEA Japan were housed in a specific-pathogen-free facility under climate-controlled conditions with a 12-h light/dark cycle and were provided with water and standard diet *ad libitum*. All animal experiments were performed according to the guidelines issued by the Institutional Animal Care and Use Committee in accordance with National Institute of Health (NIH) guidelines. Animals were treated according to the guidelines and regulations of the Laboratory Animal Research Center at Sungkyunkwan University School of Medicine.

### Ovariectomy and estrogen pellet insertion

Experiments were performed in three groups: sham-operated (sham (ovary intact)), ovariectomized and ovariectomized + E2. Seven-week-old female SKG mice underwent an ovariectomy (OVx) or a sham operation under anesthesia 2 weeks before arthritis induction. At 8 weeks of age, slow-releasing pellets of E2 (0.72 mg, 60 days release, Innovative Research, Sarasota, FL, USA) were implanted subcutaneously on the neck scruff of mice in the E2-treated group [19]. For these mice, a 0.5-cm incision was made in the loose skin of each mouse’s neck, and a small pocket was bluntly dissected caudolaterally. The pellet was installed in the pocket using tweezers. The incision was subsequently closed with a suture.

### Induction of arthritis and scoring of clinical signs

Arthritis was induced 2 weeks after ovariectomy or 1 week after E2 pellet implantation. Zymosan A (Sigma, St Louis, MO, USA) was suspended in PBS and incubated for 10 min in boiling water. Then the zymosan A solution was injected intraperitoneally into 9-week-old mice in each of the three groups (3 mg/mouse): sham (ovary intact), ovariectomized and ovariectomized + E2 groups. ^18^F-fluorodeoxyglucose (^18^F-FDG) small-animal positron emission tomography (PET)/computed tomography (CT) and cytokine analysis were also compared with the wild-type control group. Clinical scores were monitored following a previously published system [17]: 0, no swelling or redness; 0.1, swelling or redness of the digits; 0.5, mild swelling and/or redness of the wrists or ankle joints; and 1, severe swelling of the larger joints. Scores for the affected joints were totaled for each mouse. The maximum possible score was 6. We defined tail changes as “present” when a mouse developed redness, swelling, hair loss, or bumps along the tail. The presence of periocular changes surrounding the eyeball (blepharitis) was also assessed. Clinical scores were monitored weekly, and mice were sacrificed 8 weeks after zymosan injection.

### Measurement of serum estrogen

Serum levels of E2 were measured using a Mouse/Rat Estradiol enzyme-linked immunosorbent assay (ELISA) kit (Calbiotech, CA, USA) according to the manufacturer`s instructions.

### ^18^F-FDG PET/CT acquisition and image analysis

Mice were fasted for 5 h before micro-PET/CT studies. A 1.8-MBq injection of ^18^F-FDG mixed with isotonic saline in a total volume of 150 μL was administered through a tail vein. Imaging was performed 1 h later under isoflurane anesthesia without respiratory gating. Micro-PET images of mice were acquired using an Inveon micro-PET/CT scanner (Siemens Medical Solutions, Malvern, PA, USA) at the Center for Molecular and Cellular Imaging, Samsung Biomedical Research Institute (Seoul, Korea). Acquisition of non-enhanced CT images was followed by PET imaging. Micro-PET images obtained were reconstructed using 3D-ordered subset expectation maximization and then processed using Siemens Inveon Research Workplace 4.1. Three-dimensional regions of interest (ROIs) were drawn over the joints using a threshold range of 70–100% of the maximum intensity, and the average signal level in the ROIs was measured. In the case of the intestine, the ROIs were measured in the abdomen excluding the kidneys and bladder, and percentage injected dose per gram (%ID/g) was measured at a threshold value of 70–100%. Image counts/pixel/s were converted to radioactivity concentrations that were then corrected for injected radioactivity. Researchers were blinded to all clinical data, and ROIs were measured by a technician. Results are expressed as the mean ± SD %ID/g.

### Histopathological examination

Mice were anesthetized and euthanized after 8 weeks of zymosan injection. Mouse joint tissues were fixed in 10% formalin, decalcified in ethylenediamine tetraacetic acid (EDTA), and embedded in paraffin. Sections were deparaffinized using xylene, dehydrated in a graded series of alcohol solutions, and stained with hematoxylin and eosin (H&E). Histologic features of the mouse joints were scored as described previously [18]: 1–4, where 1 = few infiltrating immune cells, 2 = 1–2 small patches of inflammation, 3 = inflammation throughout the joint, and 4 = inflammation in soft tissue/entheses/fasciitis. Histologic features of the tail were scored on a scale of 1–4, where 1 = few infiltrating immune cells, 2 = mild inflammation of the discs or along the vertebrae (0–30% of discs), 3 = inflammation of the discs and/or along the vertebrae (30–70% of discs), and 4 = inflammation in > 70% of the discs and along the vertebrae. Microscopic gut inflammation was assessed. Intestines were scored 1 for the presence and 0 for the absence of ileitis or colitis.

### RNA isolation and QuantiGene 2.0 Plex assay

Total RNA was extracted from the hind paws and forepaws using a total RNA kit (QuantiGene sample processing kit) according to the manufacturer’s protocol. Target hybridization and signal amplification were performed according to the manufacturer’s protocol for fresh tissues (QuantiGene 2.0 Plex assay). Signal was detected using a Luminex 100/200 system and reported as median fluorescence intensity (MFI), which is proportional to the number of target RNA molecules present in the sample. Gene expression was first calculated by determining the average signal (MFI) for all genes, and average background signals were subtracted for each gene. Levels of gene expression were then determined by dividing each test gene signal (background subtracted) by the normalization gene signal (background subtracted). Next, fold changes were determined by dividing the normalized value for zymosan-treated samples by the normalized values for the healthy controls. Each sample was assayed using technical duplicates. Target genes were tumor necrosis factor (TNF), IL-6, interferon-γ (IFNγ), IL-4, IL-17A, IL-23, Dkk1, and sclerostin (SOST).

### Statistical analyses

One-way analysis of variance (ANOVA) was used to compare normally distributed means. Two-way ANOVA was used to analyze treatment effects on clinical scores over time. Tukey’s post hoc test was performed to compare multiple means. Results are presented as mean ± standard error of the mean (SEM). All analyses were performed using SPSS software, version 19.0 (SPSS, Inc., Chicago, IL, USA) and GraphPad Prism (version 5.0, GraphPad Software, San Diego, CA, USA).

## Results

### Estrogen treatment suppressed arthritis development in SKG mice

**Fig. 1:**
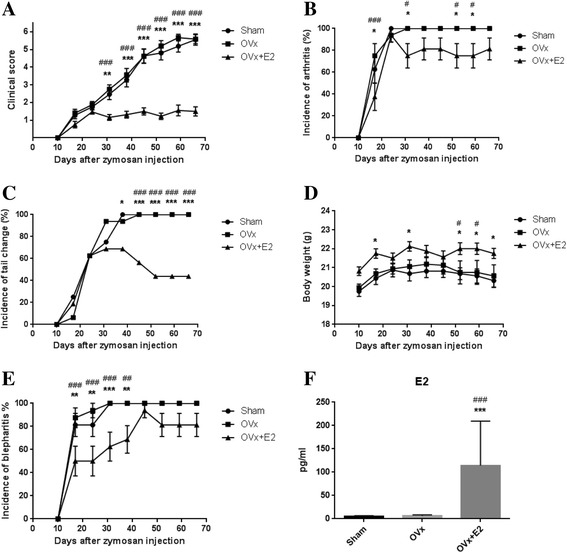
17β-estradiol (E2) treatment suppressed arthritis development in SKG mice. Clinical scores were markedly suppressed in E2-treated mice compared with mice in the sham-operated (Sham) and ovariectomized (OVx) groups after 31 days of zymosan injection. Clinical scores (**a**), the incidence of arthritis (**b**), the incidence of tail changes (**c**), body weight (**d**), and the incidence of blepharitis (**e**) in the three groups. Serum levels of E2 were measured by ELISA (**f**). Values are mean ± SEM (n = 16 mice per group); *P < 0.05, **P < 0.01, ***P < 0.001 (sham vs. OVx + E2); ^#^ *P* < 0.05; ^##^ *P* < 0.01; ^###^ *P* < 0.001 (OVx vs. OVx + E2)

The effect of estrogen on arthritis development in female SKG mice was examined. Weekly observations revealed that clinical scores were markedly suppressed in E2-treated mice compared with the sham and ovariectomized groups (Fig. 1a). The onset of arthritis was similar among groups until 24 days after zymosan injection. From 31 days after zymosan injection, arthritis was more aggravated and clinical scores had increased up to 6 points (maximal score) in the sham and ovariectomized groups. However, clinical scores had not increased after 31 days in the E2-treated mice. Clinical scores were significantly lower in E2-treated mice than those in the other two groups from 31 days after zymosan injection until the last observation. The incidence of arthritis was lower in the E2-treated group (Fig. 1b). The incidence of tail changes (Fig. 1c) was higher in the sham and ovariectomized groups than in the E2-treated group. Body weight was significantly higher in the E2-treated group than in the other two groups (Fig. 1d). Mice in the sham and ovariectomized groups achieved a near 100% incidence of blepharitis at an early stage after zymosan injection (Fig. 1e). Mean serum E2 in E2-treated mice was significantly higher than in the other groups (Fig. 1f). There were no significant differences in serum E2 between the sham and ovariectomized groups.

### E2 treatment reduced ^18^F-FDG uptake using micro-PET/CT imaging

**Fig. 2:**
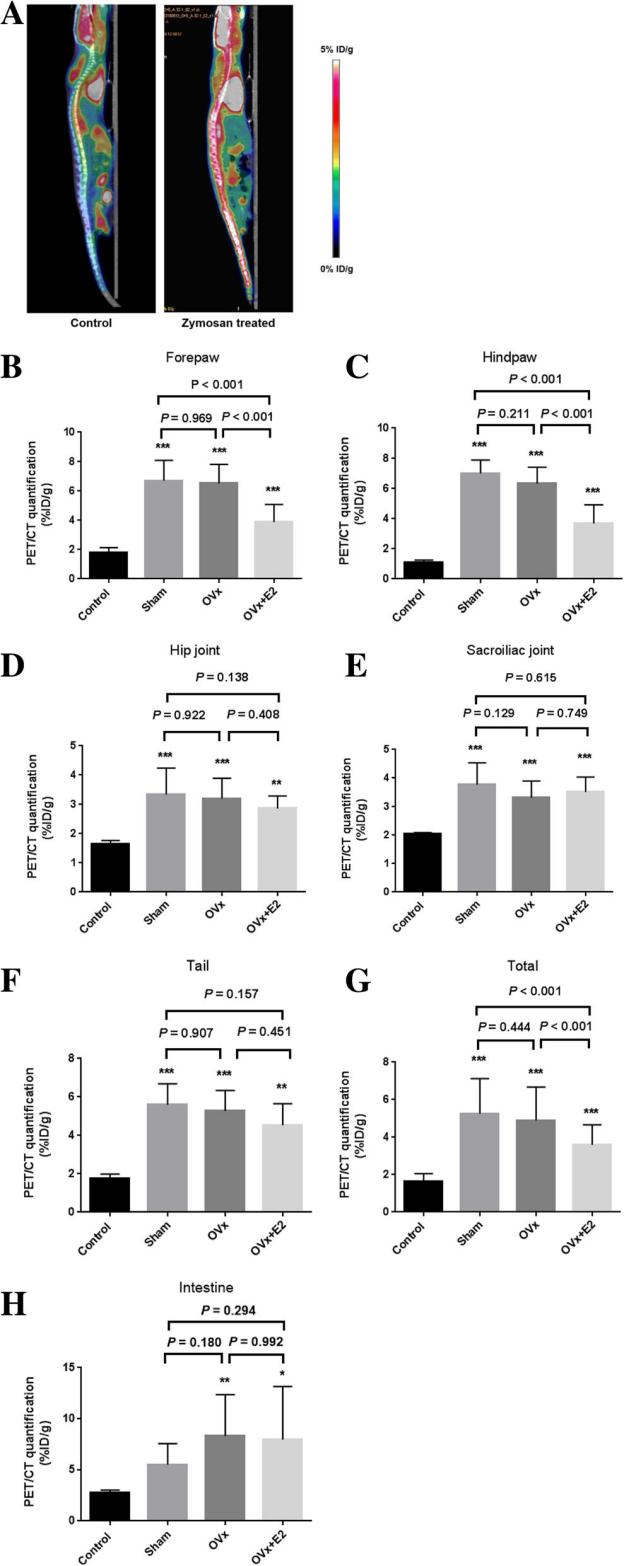
Quantitative measurement of ^18^F-fluorodeoxyglucose (^18^F-FDG) uptake. Visualization of ^18^F-FDG uptake in the spine of a wild-type (control) mouse and a mouse 8 weeks after zymosan injection (zymosan-treated) (**a**). Quantification of regions of interest (ROIs) in control, sham-operated (Sham), ovariectomy (OVx), and OVx + 17β-estradiol (OVx + E2) groups. **b**-**g** Forepaw (**b**), hind paw (**c)**, hip joint (**d**), sacroiliac joint (**e**), tail (**f**), total of ROIs of five joints (**g**), and intestine (**h**). Values are mean ± SEM, n = 12 mice per group except for the wild-type control group (*n* = 2); *P < 0.05, **P < 0.01, ***P < 0.001 versus controls. PET/CT positron emission tomography/computed tomography

Figure 2a shows ^18^F-FDG uptake in mice spines using micro-PET/CT imaging. The zymosan-treated group had higher ^18^F-FDG uptake and loss of lordosis than the normal wild-type control group. Quantification of the ROIs of the control, sham, ovariectomy, and ovariectomy + E2 groups showed that the amount of ^18^F-FDG uptake was significantly higher in the sham, ovariectomized, and E2-treated groups than in wild-type controls. The E2-treated group had significantly lower ^18^F-FDG uptake than the sham and ovariectomized groups in both forepaws (Fig. 2b) and hind paws (Fig. 2c). There were no significant differences in the hip joints (Fig. 2d), sacroiliac joints (Fig. 2e), or tail (Fig. 2f) among the sham, ovariectomized, and E2-treated groups. In a total of five joint ROIs, ^18^F-FDG uptake was significantly lower in the E2-treated group than in the sham and ovariectomized groups (Fig. 2g). ^18^F-FDG uptake was not significantly different between the sham and ovariectomized groups. In addition, ^18^F-FDG uptake in the intestine was significantly higher in the ovariectomized and E2-treated groups than the wild-type control group (Fig. 2h). There was no difference between the sham, ovariectomized, and E2-treated groups.

### Histologic scores for arthritis and spondylitis were lower in E2-treated mice

**Fig. 3:**
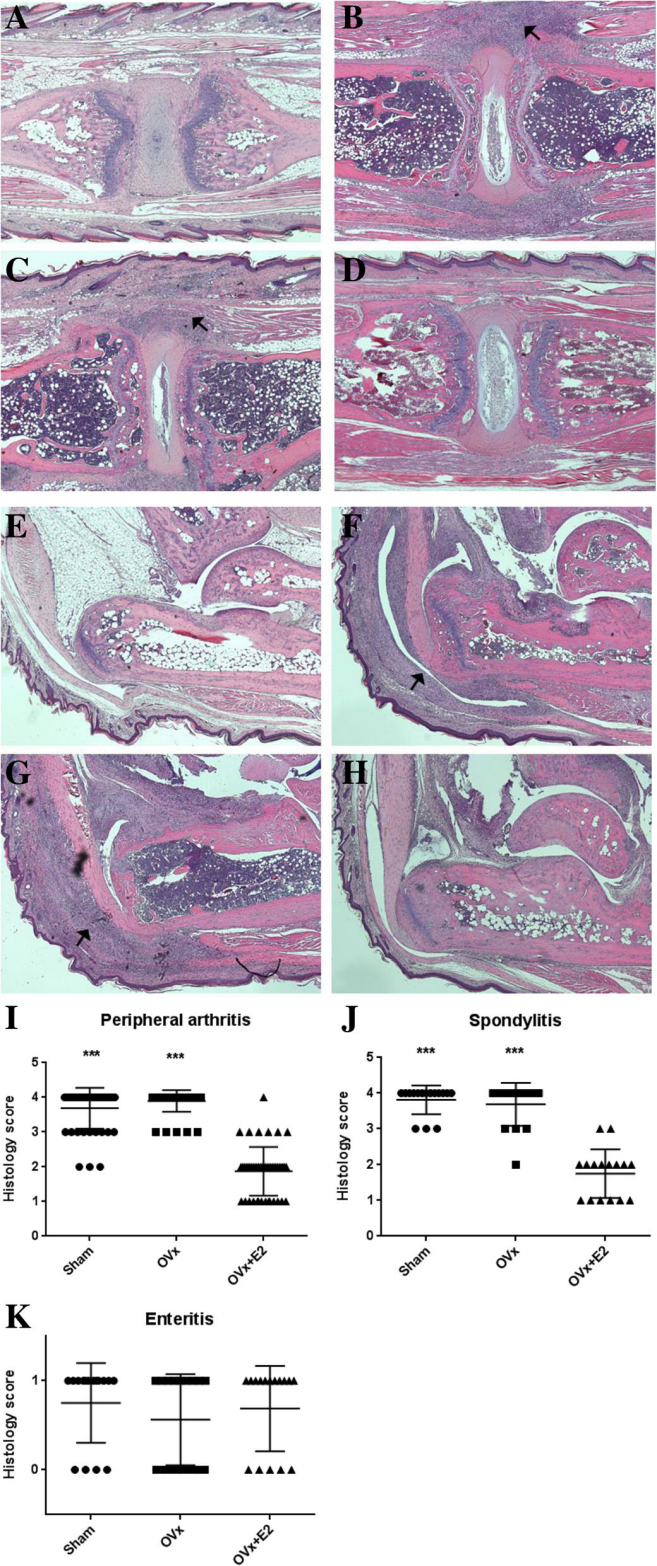
The effect of estrogen on microscopic inflammation. **a**-**d** The intervertebral area of the wild-type control (**a**), sham-operated (Sham) (**b**), ovariectomized (OVx) (**c**), and 17β-estradiol (E2)-treated (OVx + E2) mice (**d**). Arrows in **b** and **c** indicate inflammatory cell infiltration of the intervertebral disc area. **e**-**f** Achilles tendon of the normal control (**e**), sham (**f**), ovariectomized (**g**), and E2-treated mice (**h**). Arrows in **f** and **g** indicate inflammatory cell infiltration in the Achilles tendon. Inflammatory cell infiltration was not evident in E2-treated mice. Staining with H&E. Original magnification × 40. Histologic severity scores for peripheral joints affected by peripheral arthritis, including the forepaws, hind paws, ankle joints (**i**), tail (**j**), and intestine (**k**). Symbols represent individual mice, horizontal lines represent the mean, and whiskers represent the SEM; *n* = 16 mice per group except for the wild-type control (n = 2); *P < 0.05, **P < 0.01, ***P < 0.001 versus controls

Histologic analysis of the mice at 8 weeks after zymosan injection was performed. Inflammation of the ankles, Achilles tendons, plantar fascia, and area around the intervertebral discs was observed. As an extra-articular manifestation, infiltration of inflammatory cells was also seen in the skin of the ears, periorbital soft tissues, and intestines. Figure 3 shows the histologic features of spondylitis and Achilles tendinitis. Inflammation of the intervertebral discs in the tail was prominent in the sham (Fig. 3b) and ovariectomized mice (Fig. 3c). E2-treated mice (Fig. 3d) had a few inflammatory cell infiltrations compared to the wild-type controls (Fig. 3a), but the degree of inflammation was milder than in the sham and ovariectomized mice. The Achilles tendons of both the sham (Fig. 3f) and ovariectomized groups (Fig. 3g) were severely inflamed. E2-treated mice (Fig. 3h) had inflammation compared to the wild-type controls (Fig. 3e), but the degree of inflammation was mild compared with the other two groups. Histologic scores for peripheral arthritis (Fig. 3i) and spondylitis (Fig. 3j) were significantly lower in E2-treated mice than in mice in the other groups. Most of the mice in the sham and ovariectomized groups had very severe inflammation. There was no difference between the groups in the presence of enteritis (Fig. 3k). About 70% of mice in the sham, ovariectomized, and E2-treated groups had inflammation of the gut.

### Expression of TNFɑ, IFN-γ, and IL-17A was significantly reduced in E2-treated mice

**Fig. 4:**
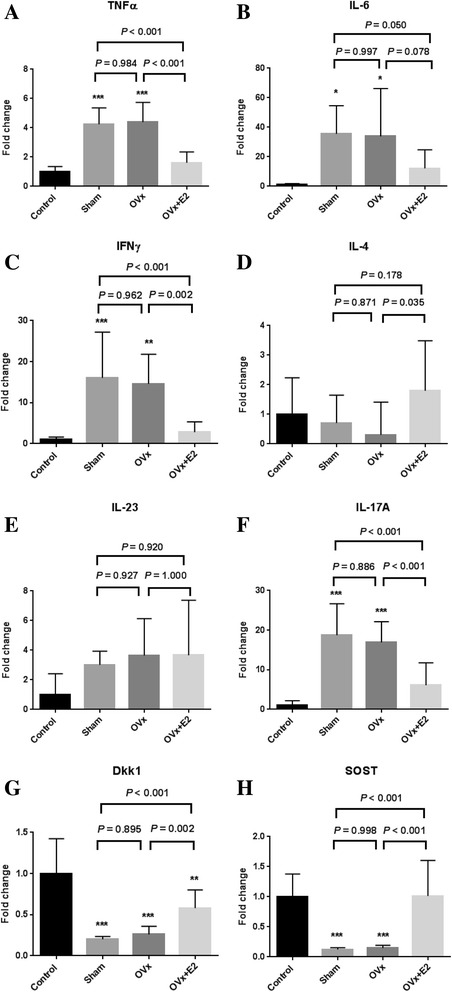
Local mRNA expression in the hind paws and forepaws was analyzed using the QuantiGene 2.0 Plex assay. Fold change in gene expression was compared to that in wild-type controls. **a**-**h** TNFɑ (**a**), IL-6 (**b**), interferon-γ (IFN-γ) (**c**), IL-4 (**d**), IL-23 (**e**), IL-17A (**f**), dickkopf-related protein 1 (Dkk1) (**g**), and sclerostin (SOST) (**h**). Data are expressed as the average fold change relative to the wild-type control group. Values are mean ± SEM; *n* = 12 mice per group except for the wild-type control (*n* = 2); *P < 0.05, **P < 0.01, ***P < 0.001, versus controls. Sham = sham-operated mice, OVx = ovariectomized mice, OVx + E2 = 17β-estradiol-treated ovariectomized mice

Local gene expression in the tissues of the hind paws and forepaws, which were harvested at 8 weeks after zymosan injection, was analyzed using the QuantiGene 2.0 Plex assay (Fig. 4). Expression of TNF, IL-6, IL-17A, and IFN-γ was significantly increased in both the sham and ovariectomized groups compared with the wild-type control group. Interestingly, E2 treatment significantly reduced the gene expression of TNF, IL-6, IFN-γ, and IL-17A compared with expression of these genes in the sham and ovariectomized groups (Figs. 4a-c and f). Expression of IL-4 was significantly higher in the E2-treated group than in the ovariectomized group (Fig. 4d). Expression of IL-23 was not significantly different among groups (Fig. 4e). Wnt inhibitors, Dkk1 and SOST, were significantly downregulated in both the sham and ovariectomized groups (Figs. 4g and h). The E2-treated group had significantly increased expression of Dkk1 and SOST compared with the sham and ovariectomized groups.

## Discussion

We investigated the role of estrogen in the disease activity of SpA. We found that estrogen suppressed the development of arthritis in SpA, using an animal model. The E2-treated group had significantly suppressed arthritis compared with both the ovariectomized and the sham groups. There was no difference between the ovariectomized and sham groups. We found that arthritis was induced in almost all female mice with normal ovaries, and the clinical score was almost 6 (the maximum score). We could not assess the difference between mice in the ovariectomized group and mice that received a physiologic dose of estrogen (the sham group) because female mice with normal ovaries (the sham group) already had zymosan-induced severe arthritis. A high dose of E2 pellets was used (72 mg, 60 days release), which was higher than the physiologic concentration [19]. The different responses between the E2-treated group and the sham group were likely due to the different estrogen doses received by these two groups.

The current study demonstrated that E2-treated mice had decreased expression of TNF in the joint tissue. These results support the hypothesis that estrogen inhibits the production of TNF [20]. E2 has been shown to downregulate TNF production and reduce the severity of experimental autoimmune encephalomyelitis in cytokine knockout mice [21]. Estrogen-deficient female rats are also reported to have higher bioactive serum TNF than estrogen-supplemented animals [22]. An in vitro study showed that E2 treatment downregulates cytokine-induced TNF gene expression [23]. Considering that TNF is a key cytokine in the pathogenesis of SpA, the results of the current study indicate that transcription of TNF might be regulated by estrogen in patients with SpA.

The current study demonstrated that E2 treatment significantly reduced the gene expression of IFN-γ, whereas the expression of IL-4 increased significantly in the joint tissues in response to E2 treatment. This corresponds well with recent data from a mouse model of autoimmune encephalomyelitis [24]. In that study, high and medium doses of estrogen increased the production of IL-4, IL-10, and transforming growth factor (TGF)-β, whereas estrogen treatment reduced the production of IFN-γ, IL-17, and IL-6. Estrogen triggers a shift in the Th1/Th2 balance toward Th2 production [25]. Thus, in Th1-mediated autoimmune diseases such as RA, symptoms often decrease during pregnancy and flare or initially develop in the postpartum period. In contrast, Th2-mediated diseases such as systemic lupus erythematosus (SLE) often flare during pregnancy [26]. Estrogen also inhibits Th17 cell production. In ovariectomized DBA/1 mice with established collagen-induced arthritis (CIA), E2 treatment reduces the severity of arthritis and results in fewer Th17 cells in the joints compared with controls [27]. Tyagi et al. reported that transcription factors that promote Th17 cell differentiation and IL-17 levels are increased in ovariectomized mice, and that these effects are reversed by E2 supplementation [13]. Another study also showed that E2 inhibits arthritis and reduces IL-17-producing γδT cells in the joints of CIA mice [28]. It is well known that the Th17 axis is overactive in patients with AS [29, 30], and the percentage of Th17 cells is significantly lower in nr-axSpA than in AS. Our data suggest that E2 modulates cells with IL-17-producing capacity during arthritis development in SpA.

The mechanism of the relationship between estrogen and T cell differentiation is unknown. In cross-sectional studies, testosterone levels have been found to be similar in patients with AS and controls, but serum dehydroepiandrosterone sulfate (DHEAS) and 17α- hydroxyprogesterone were more elevated in male patients with AS than in male controls [31–33]. The gene for the enzyme, 21-hydroxylase, is located close to the human leukocyte antigen (HLA)-B locus on the short arm of chromosome 6. HLA-B27 might serve as a marker of partial 21-hydroxylase deficiency, resulting in high DHEAS and 17α-hydroxyprogesterone levels. A proinflammatory Th1 phenotype might be enhanced by increased DHEAS, whereas estrogen and progesterone suppress the Th1 phenotype [12]. β-catenin/TCF promotes differentiation of Th2 from naïve CD4 T cells and prevents the differentiation of Th17/Th1. Estrogen might be involved in T cell differentiation via the Wnt/β-catenin pathway [34]. The relationship between estrogen and T cell differentiation in SpA has not been investigated; therefore, further study is needed to determine the role of estrogen in T cell differentiation in SpA.

In the current study, we also evaluated the role of Wnt inhibitors in the disease activity of SpA. The German Spondyloarthritis Inception Cohort (GESPIC) study reported that patients with lower Dkk1 levels were more prone to develop syndesmophytes than those with higher levels [35]. In the present study, expression of Dkk1 was decreased in zymosan-treated mice compared with controls. In addition, expression of Dkk1 was significantly increased in E2-treated mice compared with the ovariectomized and sham groups. This corresponds well with the previous finding that expression of Dkk1 and SOST was reduced in the spine of a proteoglycan-induced spondylitis (PGISp) mouse model of SpA, compared with controls [36]. SOST is another Wnt inhibitor that is a potent suppressor of bone formation, and inhibits bone morphogenic protein. The absence of SOST expression results in increased bone formation and bone mass [37]. In the current study, SOST levels were significantly lower in all of the zymosan-treated groups than in the control group, and SOST levels were higher in the E2-treated group than the other groups. In the cohort study, devenir des spondyloarthrites indifférenciées récentes (DESIR), SOST was significantly lower in patients with SpA than in healthy controls [38]. In addition, SOST was significantly lower in patients with SpA than in control patients, whereas SOST was increased in patients with RA compared with controls [39, 40]. We therefore evaluated the expression of Wnt inhibitors in the SKG mouse model and demonstrated that estrogen inhibited Wnt signaling in SpA. Considering that blockade of Wnt inhibitors induced the fusion of sacroiliac joints and increased bone formation [37, 41], estrogen might be used as a therapeutic target to control the Wnt pathway.

This study had several limitations. First, uterine weights were not measured; rather, we measured the concentration of E2 in mouse serum. The mean concentration of E2 was significantly higher in E2-treated mice than in mice in the other groups, but there was no significant difference between E2 levels in the sham and ovariectomized groups. Inflammation affects ovulation and hormone production. Moreover, 50% of cases of premature ovarian failure have been ascribed to autoimmune disease [42]. Considering that mice in the sham group had severe inflammation in the joints, intestines, and skin at the time of sacrifice, estrogen production might have been decreased due to ovarian failure induced by systemic inflammation. Second, gene expression in the tail was not quantified. However, considering that the incidence of arthritis and the degree of inflammation in the peripheral joints were clinically and histologically closely tied to those of the tail joints, the gene expression patterns found in the joint tissue of the hind paws and forepaws likely reflected those in the tail joints. The strength of this study is that it is one of the few studies to evaluate the role of estrogen in disease activity in SpA. To the best of our knowledge, this is the first study to demonstrate the inhibitory effect of estrogen on SpA, using an animal model of SpA. Furthermore, our results suggest that estrogen might be an effective therapeutic approach to treating SpA. However, hormone treatment has various adverse effects, such as increased risk of breast cancer, endometrial cancer, and thromboembolism. Therefore, selective estrogen receptor modulators (SERMs) were developed to prevent their side effects. Recent studies reported that SERMs inhibit joint inflammation and osteoporosis in female CIA animal models [43, 44]. Further experiments are needed to evaluate the effects of SERMs in the SpA mouse model.

## Conclusions

Estrogen suppressed arthritis development in an SpA mouse model. Results of this study suggest that estrogen has an anti-inflammatory effect on the SpA manifestations in the SKG arthritis model. Further study is warranted to determine the molecular mechanisms of estrogen in the pathogenesis of SpA and to evaluate if estrogen is an effective therapeutic treatment option for SpA.

## Abbreviations

^18^F-FDG: ^18^F-fluorodeoxyglucose
%ID/g: Percentage injected dose per gram
ANOVA: Analysis of variance
AS: Ankylosing spondylitis
ASAS: Assessment of SpondyloArthritis International Society
CIA: Collagen-induced arthritis
CRP: C-reactive protein
CT: Computed tomography
DC: Dendritic cell
DHEAS: Dehydroepiandrosterone sulfate
Dkk1: Dickkopf-related protein 1
E2: 17β-estradiol
EDTA: Ethylenediamine tetraacetic acid
ELISA: Enzyme-linked immunosorbent assay
ER: Estrogen receptor
H&E: Hematoxylin and eosin
HLA: Human leukocyte antigen
IFN: Interferon
IL: Interleukin
MFI: Median fluorescence intensity
MRI: Magnetic resonance imaging
nr-axSpA: Non-radiographic axial spondyloarthritis
OVx: Ovariectomy
PBS: Phosphate-buffered saline
PET: Positron emission tomography
RA: Rheumatoid arthritis
ROI: Region of interest
SEM: Standard error of the mean
SERM: Selective estrogen receptor modulator
SOST: Sclerostin
SpA: Spondyloarthritis
Th: T helper
TNF: Tumor necrosis factor

## References

[1] Lee W, Reveille JD, Davis JC, Learch TJ, Ward MM, Weisman MH (2007). Are there gender differences in severity of ankylosing spondylitis? Results from the PSOAS cohort. Ann Rheum Dis..

[2] Rudwaleit M, van der Heijde D, Landewe R, Listing J, Akkoc N, Brandt J (2009). The development of Assessment of SpondyloArthritis international Society classification criteria for axial spondyloarthritis (part II): validation and final selection. Ann Rheum Dis..

[3] Sieper J, van der Heijde D (2013). Review: Nonradiographic axial spondyloarthritis: new definition of an old disease?. Arthritis Rheum..

[4] Oostveen J, Prevo R, den Boer J, van de Laar M (1999). Early detection of sacroiliitis on magnetic resonance imaging and subsequent development of sacroiliitis on plain radiography. A prospective, longitudinal study. J Rheumatol.

[5] Rudwaleit M, Haibel H, Baraliakos X, Listing J, Marker-Hermann E, Zeidler H (2009). The early disease stage in axial spondylarthritis: results from the German Spondyloarthritis Inception Cohort. Arthritis Rheum..

[6] Kiltz U, Baraliakos X, Karakostas P, Igelmann M, Kalthoff L, Klink C (2012). Do patients with non-radiographic axial spondylarthritis differ from patients with ankylosing spondylitis?. Arthritis Care Res (Hoboken).

[7] Dougados M, d'Agostino MA, Benessiano J, Berenbaum F, Breban M, Claudepierre P (2011). The DESIR cohort: a 10-year follow-up of early inflammatory back pain in France: study design and baseline characteristics of the 708 recruited patients. Joint Bone Spine..

[8] Jeong H, Yoon JY, Park EJ, Hwang J, Kim H, Ahn JK (2015). Clinical characteristics of nonradiographic axial spondyloarthritis in Korea: a comparison with ankylosing spondylitis. Int J Rheum Dis..

[9] Ortega Castro R, Font Ugalde P, Castro Villegas MC, Calvo Gutierrez J, Munoz Gomariz E, Zarco Montejo P (2013). Different clinical expression of patients with ankylosing spondylitis according to gender in relation to time since onset of disease. Data from REGISPONSER. Reumatol Clin.

[10] Jimenez-Balderas FJ, Tapia-Serrano R, Madero-Cervera JI, Murrieta S, Mintz G (1990). Ovarian function studies in active ankylosing spondylitis in women. Clinical response to estrogen therapy. J Rheumatol.

[11] Nalbandian G, Kovats S (2005). Estrogen, immunity & autoimmune disease. Curr Med Chem – Immun, Endoc & Metab Agents..

[12] Giltay EJ, van Schaardenburg D, Gooren LJ, Popp-Snijders C, Dijkmans BA (1999). Androgens and ankylosing spondylitis: a role in the pathogenesis?. Ann NY Acad Sci..

[13] Tyagi AM, Srivastava K, Mansoori MN, Trivedi R, Chattopadhyay N, Singh D (2012). Estrogen deficiency induces the differentiation of IL-17 secreting Th17 cells: a new candidate in the pathogenesis of osteoporosis. PLoS ONE..

[14] Lelu K, Laffont S, Delpy L, Paulet PE, Perinat T, Tschanz SA (2011). Estrogen receptor alpha signaling in T lymphocytes is required for estradiol-mediated inhibition of Th1 and Th17 cell differentiation and protection against experimental autoimmune encephalomyelitis. J Immunol..

[15] Diarra D, Stolina M, Polzer K, Zwerina J, Ominsky MS, Dwyer D (2007). Dickkopf-1 is a master regulator of joint remodeling. Nat Med..

[16] MacDonald BT, Joiner DM, Oyserman SM, Sharma P, Goldstein SA, He X (2007). Bone mass is inversely proportional to Dkk1 levels in mice. Bone..

[17] Sakaguchi N, Takahashi T, Hata H, Nomura T, Tagami T, Yamazaki S (2003). Altered thymic T-cell selection due to a mutation of the ZAP-70 gene causes autoimmune arthritis in mice. Nature..

[18] Ruutu M, Thomas G, Steck R, Degli-Esposti MA, Zinkernagel MS, Alexander K (2012). beta-glucan triggers spondylarthritis and Crohn's disease-like ileitis in SKG mice. Arthritis Rheum.

[19] Ingberg E, Theodorsson A, Theodorsson E, Strom JO (2012). Methods for long-term 17beta-estradiol administration to mice. Gen Comp Endocrinol..

[20] Roggia C, Gao Y, Cenci S, Weitzmann MN, Toraldo G, Isaia G (2001). Up-regulation of TNF-producing T cells in the bone marrow: a key mechanism by which estrogen deficiency induces bone loss in vivo. Proc Natl Acad Sci USA.

[21] Ito A, Bebo BF, Matejuk A, Zamora A, Silverman M, Fyfe-Johnson A (2001). Estrogen treatment down-regulates TNF-alpha production and reduces the severity of experimental autoimmune encephalomyelitis in cytokine knockout mice. J Immunol..

[22] Arenas IA, Armstrong SJ, Xu Y, Davidge ST (2005). Chronic tumor necrosis factor-alpha inhibition enhances NO modulation of vascular function in estrogen-deficient rats. Hypertension..

[23] Srivastava S, Weitzmann MN, Cenci S, Ross FP, Adler S, Pacifici R (1999). Estrogen decreases TNF gene expression by blocking JNK activity and the resulting production of c-Jun and JunD. J Clin Invest..

[24] Haghmorad D, Salehipour Z, Nosratabadi R, Rastin M, Kokhaei P, Mahmoudi MB, et al. Medium-dose estrogen ameliorates experimental autoimmune encephalomyelitis in ovariectomized mice. J Immunotoxicol. 2016;13:885–96. 10.1080/1547691X.2016.1223768 27602995

[25] Formby B (1995). Immunologic response in pregnancy. Its role in endocrine disorders of pregnancy and influence on the course of maternal autoimmune diseases. Endocrinol Metab Clin North Am.

[26] Wilder RL (1998). Hormones, pregnancy, and autoimmune diseases. Ann NY Acad Sci..

[27] Andersson A, Stubelius A, Karlsson MN, Engdahl C, Erlandsson M, Grahnemo L (2015). Estrogen regulates T helper 17 phenotype and localization in experimental autoimmune arthritis. Arthritis Res Ther..

[28] Andersson A, Grahnemo L, Engdahl C, Stubelius A, Lagerquist MK, Carlsten H (2015). IL-17-producing gammadeltaT cells are regulated by estrogen during development of experimental arthritis. Clin Immunol..

[29] Shen H, Goodall JC, Hill Gaston JS (2009). Frequency and phenotype of peripheral blood Th17 cells in ankylosing spondylitis and rheumatoid arthritis. Arthritis Rheum..

[30] Wendling D, Cedoz JP, Racadot E, Dumoulin G (2007). Serum IL-17, BMP-7, and bone turnover markers in patients with ankylosing spondylitis. Joint Bone Spine..

[31] Giltay EJ, Popp-Snijders C, van Schaardenburg D, Dekker-Saeys BJ, Gooren LJ, Dijkmans BA (1998). Serum testosterone levels are not elevated in patients with ankylosing spondylitis. J Rheumatol..

[32] Tapia-Serrano R, Jimenez-Balderas FJ, Murrieta S, Bravo-Gatica C, Guerra R, Mintz G (1991). Testicular function in active ankylosing spondylitis. Therapeutic response to human chorionic gonadotrophin. J Rheumatol.

[33] Arniaud D, Mattei JP, Boyer J, Roux H (1998). Sex hormones in spondylarthropathies. A study in 57 patients. Rev Rhum Engl Ed.

[34] Bonewald LF, Johnson ML (2008). Osteocytes, mechanosensing and Wnt signaling. Bone..

[35] Heiland GR, Appel H, Poddubnyy D, Zwerina J, Hueber A, Haibel H (2012). High level of functional dickkopf-1 predicts protection from syndesmophyte formation in patients with ankylosing spondylitis. Ann Rheum Dis..

[36] Haynes KR, Pettit AR, Duan R, Tseng HW, Glant TT, Brown MA (2012). Excessive bone formation in a mouse model of ankylosing spondylitis is associated with decreases in Wnt pathway inhibitors. Arthritis Res Ther..

[37] Suen PK, Zhu TY, Chow DH, Huang L, Zheng LZ, Qin L (2015). Sclerostin antibody treatment increases bone formation, bone mass, and bone strength of intact bones in adult male rats. Sci Rep..

[38] Nocturne G, Pavy S, Boudaoud S, Seror R, Goupille P, Chanson P (2015). Increase in Dickkopf-1 serum level in recent spondyloarthritis. Data from the DESIR cohort. PLoS ONE..

[39] Appel H, Ruiz-Heiland G, Listing J, Zwerina J, Herrmann M, Mueller R (2009). Altered skeletal expression of sclerostin and its link to radiographic progression in ankylosing spondylitis. Arthritis Rheum..

[40] Wehmeyer C, Stratis A, Stratis A, Pap T, Dankbar B (2010). The role of the WNT inhibitor sclerostin in rheumatoid arthritis. Ann Rheum Dis..

[41] Uderhardt S, Diarra D, Katzenbeisser J, David JP, Zwerina J, Richards W (2010). Blockade of Dickkopf (DKK)-1 induces fusion of sacroiliac joints. Ann Rheum Dis..

[42] Weiss G, Goldsmith LT, Taylor RN, Bellet D, Taylor HS (2009). Inflammation in reproductive disorders. Reprod Sci..

[43] Andersson A, Bernardi AI, Nurkkala-Karlsson M, Stubelius A, Grahnemo L, Ohlsson C (2016). Suppression of experimental arthritis and associated bone loss by a tissue-selective estrogen complex. Endocrinology..

[44] Andersson A, Bernardi AI, Stubelius A, Nurkkala-Karlsson M, Ohlsson C, Carlsten H (2016). Selective oestrogen receptor modulators lasofoxifene and bazedoxifene inhibit joint inflammation and osteoporosis in ovariectomised mice with collagen-induced arthritis. Rheumatology (Oxford).

